# A systematic review of real-world healthcare resource use and costs of *Clostridioides difficile* infections

**DOI:** 10.1017/ash.2022.369

**Published:** 2023-01-17

**Authors:** Daniel C. Malone, Edward P. Armstrong, Dan Gratie, Sissi V. Pham, Alpesh Amin

**Affiliations:** 1 Strategic Therapeutics, LLC, Tucson, Arizona; 2 University of Utah College of Pharmacy, Salt Lake City, Utah; 3 University of Arizona College of Pharmacy, Tucson, Arizona; 4 AESARA, Chapel Hill, North Carolina; 5 Medicine, Business, Public Health, Nursing Science, & Biomedical Engineering, University of California–Irvine, Irvine, California; 6 Hospitalist Program, University of California–Irvine, Irvine, California

## Abstract

**Objective:**

To conduct a systematic review of published real-world evidence describing the cost and healthcare resource use for *Clostridiodes difficile* infection (CDI) in the United States.

**Methods:**

A systematic literature review was conducted searching for terms for CDI and healthcare costs. Titles of articles and abstracts were reviewed to identify those that met study criteria. Studies were evaluated to examine overall design and comparison groups in terms of healthcare resource use and cost for CDI.

**Results:**

In total, 28 articles met the inclusion criteria. Moreover, 20 studies evaluated primary CDI or did not specify, and 8 studies^1–8^ evaluated both primary CDI and recurrent (rCDI). Data from Medicare were used in 6 studies. Nearly all studies used a comparison group, either controls without CDI (N = 20) or comparison between primary CDI and rCDI (N = 7). Two studies examined costs of rCDI by the number of recurrences. Overall, the burden of CDI is significant, with higher aggregate costs for patients with rCDI. Compared with non-CDI controls, hospital length of stay increased in patients with both primary and rCDI compared to patients without CDI. Patients with primary CDI cost healthcare systems $24,000 more than patients without CDI. Additionally, 2 studies that evaluated the impact of recurrence among those patients with an index case of CDI demonstrated significantly higher direct all-cause medical costs among those with rCDI compared to those without.

**Conclusion:**

CDI, and particularly rCDI, is a costly condition with hospitalizations being the main cost driver.

*Clostridiodes difficile* infection (CDI) is the most common healthcare-associated infection in the United States and is considered an “urgent” threat by the Centers of Disease Control and Prevention.^9–11^ CDI in particular impacts females, the elderly, and the immunocompromised at higher rates.^12^ There are nearly half a million cases of CDI per year costing an estimated $5 billion.^10,13^ CDI management may require treatment across multiple healthcare settings, impacting those in the community setting, hospitalized patients, or older individuals in long-term care facilities because these patients cycle in and out of healthcare institutions with recurrent infections.^13^

Approximately 25% of patients with primary CDI have a recurrent infection after standard-of-care treatment, and those with first recurrence have a high risk of future recurrences. Recurrences are common because current therapies are effective at relieving symptoms by killing the toxin-producing bacteria or binding the toxin, but none repair gut microbiome dysfunction. In addition, *C. difficile*-targeted antibiotics do not have any effect on dormant *C. difficile* spores.^14,15^ Once a patient has a recurrence, signaling a disrupted microbiome, their risk of having subsequent recurrences rises substantially and further exacerbates the impact of CDI.^9,12^

We conducted a systematic review of that evidence to gain a better understanding of the implications of CDI on healthcare costs and resource use. We also evaluated the quantity and quality of studies that have examined the economics and healthcare use for patients with CDI and rCDI. Here, we summarize our findings, describe gaps in the literature detailing the differential impact of CDI and rCDI on patients and the US healthcare system, and identify gaps in the evidence for further investigation.

## Methods

We searched the abstracting services of PubMed, Embase, and the Cochrane Collaboration to identify research that evaluated the resource use and costs to care for patients with CDI and rCDI. Specific inclusion and exclusion criteria are listed below, but the focus of this review was on those articles reporting utilization of healthcare services and/or economic end points. Search terms and filters used for Embase and PubMed literature searches are summarized in Table 1. In addition to conducting searches of electronic abstracting databases, the references of relevant primary studies, guideline documents, published meta-analyses, and authoritative clinical reviews were examined to identify other possible articles. The Preferred Reporting Items for Systematic Reviews and Meta-Analysis (PRISMA) guidelines were followed in this analysis.^16^

**Table 1.** Literature Search Terms Utilized

Term	Embase and Medline	PubMed
*Clostridioides difficile* infection	‘clostridioides difficile’/exp OR ‘clostridioides difficile’ OR ‘clostridium infection’/exp OR ‘clostridium infection’ OR ‘clostridium difficile infection’/exp OR ‘clostridium difficile infection’ OR ‘c. diff.’ OR ‘recurrent clostridium difficile infection’/exp OR ‘recurrent clostridium difficile infection’	Using Medical Subject Headings (MeSH) terms: (“Clostridioides difficile”[Majr])
Cost	‘healthcare cost’/exp OR ‘healthcare cost’ OR economic* OR cost* OR expen* OR ((work OR productiv*) NEAR/3 (present* OR absen* OR los*)) OR ‘resource utili*’ OR ‘resource use’ OR ‘healthcare utili*’ OR hospitali* OR ‘length of stay’/exp OR ‘length of stay’ OR los OR (emergency NEAR/2 (department OR room)) OR hui OR utilit* OR (burden NEAR/5 (economic OR societ*)) OR ‘economic impact’/exp OR ‘economic impact’ OR ‘cost of illness’/exp OR ‘cost of illness’ OR ‘economic models’ OR ‘cost-effectiveness’/exp OR ‘cost-effectiveness’ OR ‘budget impact’ OR ‘health technology assessment’/exp OR ‘health technology assessment’ OR ‘productivity’/exp OR ‘human capital’/exp	Using MeSH terms: (“Health Care Economics and Organizations/analysis”[Mesh] OR “Health Care Economics and Organizations/diagnosis”[Mesh] OR “Health Care Economics and Organizations/economics”[Mesh] OR “Health Care Economics and Organizations/epidemiology”[Mesh] OR “Health Care Economics and Organizations/standards”[Mesh]) health care costs[MeSH Terms] cost of illness[MeSH Terms] budget impact[MeSH Terms]
Search limits	Articles, humans, 2010-2021, English	Humans, 2010-2021, English

Studies were eligible for inclusion if they met the following criteria: evaluated either primary or rCDI; included end points of healthcare use (eg, hospitalizations, clinic visits, etc.) or economic outcomes (eg, cost of care) for services provided in the United States; published after 2010 due to rapidly changing epidemiology of the disease, new therapies, and evolving treatment strategies; and published in English. Cost analyses from other countries were excluded because resource unit costs vary widely between countries. Economic models (eg, cost-effectiveness analyses) were beyond the scope of this review because these models are typically constructed using other published data and the focus of this review was on the primary literature.

After completing the searches from abstracting services, the titles and abstracts of identified studies were examined for relevance. Full manuscripts of potentially relevant articles were retrieved to verify eligibility and undergo data extraction. Two reviewers (D.C.M. and E.P.A.) independently conducted the literature searches and independently reviewed article titles and abstracts for inclusion in the analysis. Differences between reviewers regarding including or excluding a study were resolved by evaluation and discussion of the article by the 2 reviewers.

Each study was evaluated with respect to study design, incorporation of a comparison and/or control group, data source, end points, and sample size. For study design, we evaluated whether a comparison group was used and the use of propensity score matching (PSM). PSM is a common technique to analyze observational research because it incorporates many variables when matching cases and controls. Generally, studies using PSM have higher internal validity because the matching approach is robust and overcomes small cell sizes that plague traditional matching.

## Results

**Fig. 1.**
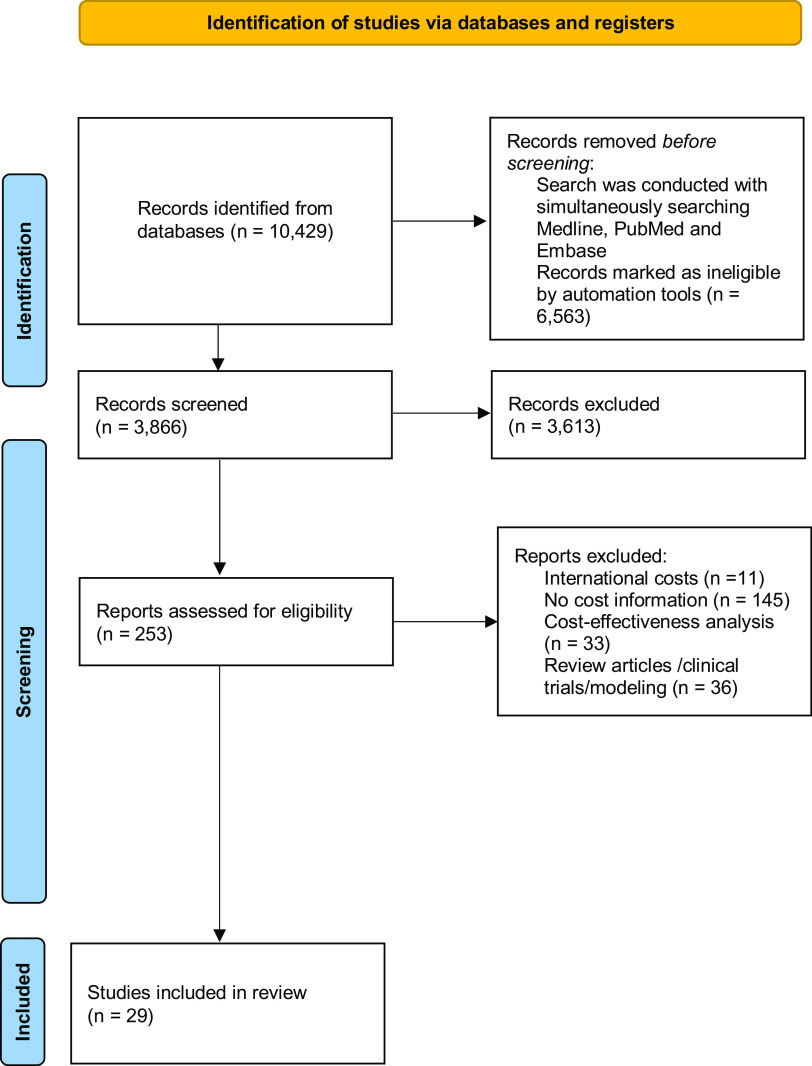
Flow diagram for systematic review processes.

Figure 1 displays the PRISMA flow diagram describing the article selection process. Initially >10,000 articles were identified, but after applying restrictions on date and language, 3,866 remained. Once title and abstracts were screened, 253 full reports were obtained and evaluated for inclusion, leading to a total of 28 published studies for review.^1–8,17–36^ Across these studies, various data sources were utilized to examine the impact of CDI and/or rCDI (Table 2). The most frequent source of data evaluated was Medicare (n = 6),^1,6,23–25,35^ followed by a variety of commercial medical and pharmacy databases (eg, Truven Health MarketScan, HealthCore Integrated Research, PharMetrics Plus, Kaiser Permanente Northern California, Cerner).^2–4,17,20,28–30^ Two publications used the Premier hospital database,^26,27^ which includes hospital services but does not capture healthcare services received beyond the hospital.

**Table 2.** Databases Used by the Identified Studies

Database or Data Source	No. of Studies Using the Source
Medicare ± Medicaid	6
Truvan Health MarketScan	3
HealthCore Integrated Research	2
Premier	2
Healthcare Cost and Utilization Project Kids	2
PharMetrics Plus	1
Partners Healthcare Network	1
University HealthSystem Consortium	1
Kaiser Permanente Northern California	1
Healthcare Cost and Utilization Project National Inpatient Sample	1
Vizient	1
New York State Health Department	1
Healthcare Cost and Utilization Project Nationwide Emergency Department Sample	1
Cerner	1
Pediatric Health Information System	1
CareFusion	1
Individual Hospital Systems	2
Simulation	1

In total, 20 studies evaluated primary CDI or did not specify, whereas 8 studies^1–8^ evaluated both primary CDI and rCDI. The study objectives and outcomes of interest varied across the articles (Table 3), but common study end points included total costs (inpatient and outpatient), hospital costs or charges, healthcare resources, hospital length of stay (LOS), patient mortality, and hospital readmission rates. We noted significant heterogeneity in the methods applied to evaluate the impact of CDI.

**Table 3.** Use of Comparator Group, Study End Points, and Study Results

Author	Year	Database	Sample Size (Cases)	Comparator Group Match	Propensity Score Match	Relevant End Points	Results
Nelson WW, Scott TA, Boules M, et al^1^	2021	Medicare	268,762	No	No	Resource use, costs	Mean total all-cause direct costs were $76,024 for no CDI recurrence and $96,517 for at least 3 CDI recurrence episodes.
Duhalde L, Lurienne L, Wingen-Heimann SM, et al^17^	2020	Truven Health MarketScan	622	Yes	No	Hospital LOS, inpatient costs	The average cost for cancer and CDI was $196,524 versus $136,365 for cancer without CDI. The average time in the hospital was 23.1 days longer with CDI.
Feuerstadt P, Stong L, Dahdal DN, et al^2^	2020	PharMetrics Plus	46,571	No	No	Costs	The mean annual total all-cause direct medical costs per CDI patient were $71,980 with no recurrence and $207,733 for those with 3 or more recurrences.
Garg SK, Obaitan I, Sarvepalli S, et al^18^	2020	Healthcare Cost and Utilization Project Nationwide Emergency Department Sample	909,236	No	No	Charges, admission, hospital LOS	There were 909,236 emergency department visits for CDI and 90% were admitted to the hospital. Charges per visit escalated to $2,900. LOS declined to 5.8 days.
Hall BR, Armijo PR, Leinicke JA, et al^19^	2019	Vizient	1,059	No	No	Mortality, hospital LOS, costs	More days from admission to surgery were associated with higher mortality, hospital LOS, infectious complications, and hospital charges.
Mollard S, Lurienne L, Heimann SM, et al^20^	2019	Truven Health MarketScan	46,097	Yes for subset	No	Hospital LOS, inpatient costs	Inpatients with CDI primary diagnosis had mean cost of $10,528 and LOS of 5.9 days. CDI as comorbidity had mean additional cost of $11,938 and added hospital LOS of 4.4 days.
Shrestha MP, Bime C, Taleban S^21^	2018	Healthcare Cost and Utilization Project national inpatient sample	587,799	No	No	Mortality, hospital LOS, costs	Hospital charges for patients with a principal diagnosis of CDI increased from $24,535 in 2004 to $35,898 in 2014. Mortality decreased from 3.6% in 2004 to 1.6% in 2014.
Zhang D, Prabhu VS, Marcela SW^3^	2018	Truven Health MarketScan	55,504	Yes	Yes	Hospital days, healthcare costs	24.8% of patients had recurrence. Average hospital days for CDI was 8.01 versus 2.81 for matched non-CDI. Average healthcare cost across all patients with primary CDI was $43,718 versus $19,513 for a matched group without CDI.
Kulayat AN, Rocourt DV, Podany AB, et al^36^	2017	Healthcare Cost and Utilization Project Kids	1,438	Yes	Yes	Hospital LOS, inpatient costs	The mean excess hospital LOS and costs attributable to CDI were 5.8 days and $12,801.
Kuntz JL, Baker JM, Kipnis P, et al^4^	2017	Kaiser Permanente Northern California	4,174	Yes	No (used “optimal matching algorithm”)	Health resources	Recurrent CDI patients had substantially higher levels of healthcare utilization than both patients with nonrecurrent CDI and patients that never had CDI.
Mehrotra P, Jang J, Gidengil C, et al^22^	2017	Healthcare Cost and Utilization Project Kids	8,527	Yes	Yes	Hospital LOS, inpatient costs	The attributable cost of CDI ranged from $1,917 to $8,317 and the increase in hospital LOS was 4 days.
Rodrigues R, Barber GE, Ananthakrishnan AN^5^	2017	Partners Healthcare Network	98	No	No	Healthcare utilization, estimated costs	84% of patients had a CDI hospitalization and total CDI-associated cost was $34,104 per patient.
Zilberberg MD, Shorr AF, Jesdale WM, et al^20^	2017	Medicare and Minimum Data Set	14,472	No	No	Hospital days, healthcare costs	Adjusted excess hospital days per patient was 20.3 and Medicare reimbursements were $12,043 in the group with recurrence.
Shah DN, Aitken SL, Barragan LF, et al^7^	2016	Single hospital	540	No	No	Recurrence, hospital LOS, costs	18% of primary CDI patients had a recurrence. Total hospital median hospital LOS and costs increased with recurrence.
Shorr AF, Zilberberg MD, Wang L, et al^23^	2016	Medicare and Medicaid	6,838	Yes	Yes	Mortality and costs	CDI was associated with near doubling of both mortality and total healthcare costs.
Yu H, Baser O, Wang U^24^	2016	Medicare, Medicaid, and Minimum Data Set	32,807	Yes	Yes	Incidence, mortality, healthcare costs	Total healthcare costs within 2 months following first CDI episode were significantly higher for CDI residents ($28,621 vs $13,644) for combined Medicare and Medicaid costs. Mortality rates higher in CDI group.
Drozd EM, Inocencio TJ, Braithwaite S, et al^25^	2015	Medicare	3,262	Yes	Yes	Mortality and costs	Patients with CDI have 1.87 times greater odds of inpatient mortality compared to those without CDI. Hospital LOS with CDI was 1.82 times greater than those without CDI. Patients with CDI had 1.16 times greater hospital cost than patients without CDI.
Gallagher JC, Reilly JP, Navalkele B, et al^26^	2015	Premier	95	No	No	90-day readmission	Recurrence occurred in 20.4% of patients on fidaxomicin and 41.3% on vancomycin. Costs estimated to be $454,800 for vancomycin and $196,200 for fidaxomicin.
Magee G, Strauss ME, Thomas SM, et al^27^	2015	Premier	84,225	Yes	Yes	Hospital LOS, inpatient costs	Hospital LOS and total costs were higher with CDI than non-CDI.
Palli SR, Broderick KC, Quimbo RA, et al^28^	2015	HealthCore Integrated Research	500	No	No	Resource use, costs	Mean cost was $35,621 (SD $100,502). Two-thirds of patients had GI or ID consultation.
Dubberke ER, Schaefer E, Reske KA, et al^8^	2014	Single hospital	421	No	No	Costs	The attributable cost of recurrent CDI was $11,631.
Campbell R, Dean B, Nathanson B, et al^29^	2013	Cerner	4,521	Yes	Yes	Hospital LOS, inpatient costs	Adjusted total hospital LOS was significantly greater with CDI in 4 of 5 subgroups. Total hospital costs were greater with CDI in patients who were aged ≥65 years and those on antibiotics.
Quimbo RA, Palli WR, Singer J, et al^30^	2013	HealthCore Integrated Research	21,177	Yes	No	Hospital LOS, inpatient costs	Incremental hospital LOS and hospitalization cost was increased for CDI across all subgroups compared to matched groups.
Sammons JS, Localio R, Xiao R, et al^31^	2013	Pediatric Health Information System	5,107	Yes	Yes	Mortality, hospital LOS, costs	In-hospital mortality was higher with CDI than matched controls. Also, mean differences in hospital LOS and total cost were higher with both community-acquired and hospital-acquired CDI than matched controls.
Tabak YP, Zilberberg MD, Johannes RS, et al^32^	2013	CareFusion	282	Yes	Yes	Mortality, hospital LOS, costs	CDI patients had higher mortality, longer hospital LOS, and higher costs.
Lipp MJ, Nero DC, Callahan MA.^33^	2012	New York State Department of Health	1,913	No	No	Charges and hospital LOS	Hospital-acquired CDI impacted both charges ($29,000 increase) and hospital LOS (12 additional hospital days).
Pakyz A, Carroll NV, Harpe SE, et al^34^	2011	University HealthSystem Consortium	10,857	Yes	No	Hospital LOS, inpatient costs	Adjusted mean cost for patients with CDI was $55,769 compared to $28,609 for controls. There was also a longer hospital LOS with cases than controls.
Stewart DB, Hollenbeak CS^35^	2011	Medicare	41,207	Yes	Yes	Mortality, hospital LOS, costs	Mean cost of hospitalization, hospital LOS, and mortality were higher with CDI than matched controls.

Note. CDI, *Clostridioides difficile* infection; GI, gastrointestinal; ID, infectious disease; LOS, length of stay; NA, not applicable; SD, standard deviation.

### Quality of CDI studies

We observed significant variability across the studies. One issue included the parameters for defining an episode of CDI. In general, studies used the *International Classification of Diseases, Ninth Revision, Clinical Modification* (ICD-9-CM) diagnosis codes. However, a few studies defined CDI based on exposure to the oral antibiotics vancomycin and fidaxomicin, which are primarily used to treat CDI.^1,26,27,34^ Another issue was the variability in the duration of time used to define another episode of rCDI, although most studies of rCDI used a relatively narrow window of time after the initial episode to define recurrence. Moreover, 2 of 8 studies defined recurrence as within 42 days of the primary CDI episode, 4 studies defined recurrence as within 56 to 60 days of the primary CDI episode, and 2 studies defined recurrence as within 84 to 90 days of the primary episode.^1–8^ Despite the various definitions, most studies evaluating recurrences did so using a relatively narrow window of time after the initial episode to account for the inherent rapidity of recurrences. Another difference across the studies was the time horizon during which resources were captured. Studies using the Agency for Healthcare Research and Quality’s data sets (n = 4) were typically cross-sectional at a point in time because tracking patients across multiple admissions was not possible. In contrast, studies using payer data, such as Medicare (n = 6) and commercial insurance claims (n=10), were able to follow patients over time. When analyzing the strength of the evidence in the identified studies, 16 (57.1%) of the 28 studies used a comparator group. Of these 16 studies, 9 (56.2%) used PSM^3,6,22,23,25,31,32,35,36^ to select controls.

### Healthcare burden of CDI and rCDI

Although some studies specifically assessed the initial CDI episode or rCDI episodes, the majority of studies compared any history of CDI (current or past) to non-CDI controls.^17,22–25,27,29–32,34–36^ Other comparisons evaluated a primary CDI episode versus rCDI^5–7^ and 1 study examined both non-CDI and rCDI compared to any CDI or a single episode.^3^ Few studies went beyond classifying patients based on the number of recurrences.

A study by Feurerstadt et al^2^ examined multiple rCDI episodes using administrative claims from ∼100 commercial insurance programs across the United States among 46,000 patients aged 18–64 years. Patients were classified as having only an initial CDI or 1, 2, or ≥3 recurrent episodes. Patients with recurrences had substantially higher total all-cause direct costs ranging from $132,000–$207,000, compared with $71,980 for those individuals without rCDI. Over a 12-month period, the percentage of persons who required hospitalization was 29% among those who did not have a CDI recurrence, whereas this percentage ranged from 53% to 69% among those with 1 to ≥3 CDI recurrences.

Nelson et al^1^ also examined the associated healthcare costs among 269,000 individuals aged ≥65 years enrolled in Medicare fee for service with an index CDI episode. In this study, 35% of patients had ≥1 episodes of rCDI over 12 months of follow-up. During this time, the all-cause direct costs (Medicare and direct payments) were $76,000 for those who did not have a subsequent recurrence compared to a range of $96,000–$99,000 for those who had ≥1 recurrences. However, unlike Feuerstadt et al, subsequent episodes were not substantially more costly than the initial episode, likely due to the effect of diagnosis-related group (DRG)–based payment rules used by Medicare. These rules are designed to reduce hospital readmissions by capping reimbursements in instances of excessive healthcare resource utilization. Thus, combined with lower reimbursement rates in general despite potential readmissions, cost associated with CDI under the Medicare program may be different than that for commercial plans.

### The impact of healthcare resource utilization of CDI and rCDI

A common theme that continued to be observed was the higher healthcare resource usage by patients with recurrent infection than primary CDI. Kuntz et al^4^ assessed the utilization of healthcare services using data from the Kaiser Foundation Health Plan. They used an “optimal matching algorithm” to identify a comparator group for CDI cases and found that 50% of patients with rCDI had at least 1 overnight hospitalization during the 12-month follow-up period compared to 38% among matched nonrecurrent CDI comparators.^4^ Patients with rCDI consistently had higher healthcare resource utilization compared to patients with primary CDI as well as patients without CDI, including a higher risk for emergency department visits, longer hospital stays, increased use of intensive care unit services, and higher 1-year mortality than patients with primary CDI.

Rodrigues et al^5^ conducted an observational study of patients with rCDI and reported that there were 15.4 outpatient office visits per patient during the year following the rCDI episode. Additionally, they noted that 94% of patients required another hospitalization and that 84% had at least 1 CDI-related hospitalization after the recurrent episode. Among hospitalized patients, 40% were discharged from the hospital to short-term rehabilitation facilities and 13% of patients died.^5^

One common outcome evaluated by 15 of the included studies was hospital LOS, which was significantly higher among patients with CDI compared to those without CDI. For example, in a recent analysis utilizing the Truven Marketscan database, Mollard et al^20^ reported that individuals who were admitted with CDI as a primary diagnosis had an average LOS of 5.9 days; for those with CDI as a secondary diagnosis, the presence of CDI as a complication increased the overall LOS by 4.4 days compared to those without CDI. In another large study, Magee et al^27^ assessed the burden of CDI in hospitalized patients using the Premier hospital database and PSM. These researchers reported that the adjusted hospital LOS was 13.2 days for patients with CDI compared to 8.5 days for patients without CDI (*P* < .01), an additional 5.7 days were attributable to CDI. This increase in LOS was also observed in studies that focused on specific populations. For example, Duhalde et al^17^ evaluated individuals with cancer and CDI and found that the average LOS was 23.1 additional days compared to cancer patients without CDI.

Three studies specifically assessed the impact of rCDI on LOS compared to a primary CDI episode. In a single-center study, Shah et al^7^ reported that recurrent CDI was associated with a median hospital LOS that was more than twice the duration associated with primary CDI (24 days vs 11 days, respectively). In another study evaluating nursing-home residents, an additional 20.3 hospital days were attributable to rCDI compared to primary CDI alone.^22^ Zhang et al^3^ utilized PSM within a large national healthcare claims database to match patients with rCDI to comparators without a recurrence and found that rCDI contributed an additional 2 hospital days over a 6-month period. Based on Zhang et al and other articles, the LOS attributable to primary CDI appears to be at least 7 days (up to 23 days for some studies), and for patients with rCDI, the cumulative LOS is typically >9 days.^3,5,17,27^

### The cost impact of CDI and rCDI

The total healthcare cost for patients who experience CDI is significant, and for those with rCDI, costs are higher compared to patients with a primary CDI episode. Zhang et al^3^ reported that patients with CDI had $24,205 (95% CI, $23,436–$25,013) higher costs compared to patients without CDI over a 6-month follow-up period.^3^ In addition, these researchers used PSM a second time to compare patients with primary CDI only to patients with one rCDI episode. Recurrent CDI contributed an additional $10,580 (95% CI, $8,849–$12,446) compared to those with primary CDI only. Yu et al^24^ studied nursing home residents and reported that 2-month follow-up costs were an additional $14,977 for patients with CDI compared to patients without CDI. Medicare paid the majority ($13,277) of this increased cost.

Further evidence concerning the burden of rCDI is provided by Shah et al,^7^ who reported that rCDI led to an additional $24,445 in healthcare costs over a 3-month period compared to CDI patients who did not experience a recurrence.^7^ A separate single-center study by Rodrigues et al^5^ estimated that the mean total rCDI-associated healthcare costs per patient was $34,104 over a 12-month period, with hospitalizations accounting for a majority of costs.^5^ Furthermore, the number of recurrences is associated with increasing costs. For example, Feuerstadt et al^13^ reported that each additional recurrence substantially increased healthcare costs by as much as $27,159 to $59,973.^13^ Notably, these studies did not report CDI-related costs nor control for potential confounding factors. In studies with 12-month follow-up, it is difficult to confidently attribute the increase in healthcare costs solely to rCDI without controlling for underlying comorbidities. As described above, Nelson et al^1^ assessed the number of episodes of CDI using Medicare data and found that the mean total all-cause direct costs were $76,024 with no CDI recurrence, $99,348 with 1 recurrent episode, $96,148 with 2 recurrent episodes, and $96,517 with 3 or more CDI recurrent episodes over a 12-month period.^1^ Also, subgroups with other comorbidities may be particularly affected by CDI. For example, Duhalde et al^17^ reported that among cancer patients with CDI, the additional cost of CDI was $60,159 compared to those without CDI.^17^

## Discussion

This systematic review found strong evidence supporting the negative impact of primary and recurrent CDI on healthcare costs and resource use, especially hospital services. Patients who develop CDI have longer lengths of stay, consume more resources, and have higher total costs compared to matched controls.^3,5,7^ In addition, CDI recurrence further increases all-cause healthcare costs, by estimates of $10,000 to $60,000, compared to a primary CDI episode, and rCDI can potentially be even more costly among patients with significant comorbidities, who are the most vulnerable population for rCDI.^3,17^

Despite the clear impact on healthcare resource utilization, there seemed to be little consensus in how cost data should be recorded and reported among studies. A number of reports used PSM to identify relevant control groups, addressing some of the potential bias observed in real-world analyses.^10^ However, 2 studies attempted to assess the impact of the number of recurrent episodes on healthcare resources and costs. The presence of a control group in many studies would facilitate comparisons between patients with and without rCDI, and any studies without this comparison should be interpreted with caution. Furthermore, observational studies that do not attempt to control for likely confounders among groups should be framed such that there are many likely factors contributing to additional healthcare utilization beyond CDI. Some of these risk factors are confounders of patients who are already more likely to regularly utilize healthcare across a variety of settings, even further enhancing the risk of rCDI.^37^ Those risk factors include a LOS for the initial hospital admission, a previous admission through the emergency department, as well as comorbidities of hypertension, moderate or severe liver disease, renal disease, dementia, cancer, and use of lipid-lowering therapy.^37^ The strongest predictor of a recurrent CDI episode is a prior history of CDI, signaling that patients with recurrence are uniquely susceptible and should be of particular focus for careful treatment and follow up.^5,6^

Several studies used treatment with antibiotics to identify patients with CDI, but exposure to these agents provides little insight into the patient’s CDI episode. Although vancomycin and fidaxomicin are recommended for primary and recurrent infections, bezlotuxumab is currently the only approved product specifically for preventing future recurrences.^38^ In patients with a history of recurrence, bezlotuxumab achieved modest improvement in recurrence rates compared to placebo (32% vs 49%, respectively); the mechanism of action is thought to be mediated through binding of *Clostridioides difficile* toxin B.^15^

This systematic literature review had several strengths and limitations. Broad search terms and literature searching strategies were incorporated to identify all previous analyses of healthcare resources and costs used in the treatment of CDI. Also, although there have been well regarded healthcare cost analyses conducted in other countries (eg, Europe and Asia), they were excluded here because resource unit costs vary widely between countries. Additionally, the window of time used to identify and define a rCDI episode differed across studies, although most of the data were generally consistent with the definitions put forth by the Infectious Diseases Society of America and Society for Healthcare Epidemiology of America guidelines, which define rCDI as a confirmed episode within 8 weeks of the primary infection, and some claims-based analyses expanded the time window to 12 weeks or more.^16,20,21,39^ Despite this limitation, this systematic review had key strengths of identifying real-world evidence publications with the rigor of comparison groups and methodologies including PSM to demonstrate the economic implications of primary and rCDI to a range of health systems.

In conclusion, CDI is a costly disease, with hospitalizations being the main cost driver. Recurrent CDI is associated with incremental cost increases across all facets of medical care, and more studies are needed to fully understand the burden of rCDI.
